# Retraction: Hsp90β is involved in the development of high salt-diet-induced nephropathy via interaction with various signalling proteins

**DOI:** 10.1098/rsob.210209

**Published:** 2021-08-04

**Authors:** Shi-hai Yan, Ning-wei Zhao, Wei-min Jiang, Xin-tong Wang, Si-qi Zhang, Xuan-xuan Zhu, Chun-bing Zhang, Yan-hong Gao, Feng Gao, Fu-ming Liu, Zhu-yuan Fang

*Open Biol.* **6**, 150159. (Published online 1 April 2016). (doi:10.1098/rsob.150159)

The *Editor-in-Chief* in agreement with the Publisher has retracted this article. Following an investigation, we have found that in figures 3, 5, 6 and 7 of the manuscript, there appears to be multiple duplicated bands and duplicated backgrounds in the western blot images. In further review of the images, the figures show clear signs of manipulation and the authors have not been able to supply the raw data as requested, therefore, the data reported in this article are unreliable. The authors have responded and acknowledge the misuse of the western blot images.

The image integrity standards and policies for the journal can be found here: https://royalsocietypublishing.org/doi/10.1098/rsob.200165.

Prof. Jon Pines FRS *Editor-in-Chief*, Open Biology

Dr Vagner Antunes Associate Editor, Open Biology

